# Recycled Polyester Geosynthetic Influence on Improvement of Road and Railway Subgrade Bearing Capacity—Laboratory Investigations

**DOI:** 10.3390/ma14237264

**Published:** 2021-11-27

**Authors:** Konrad Malicki, Jarosław Górszczyk, Zuzana Dimitrovová

**Affiliations:** 1 Faculty of Civil Engineering, Cracow University of Technology, 31-155 Cracow, Poland; kmalicki@pk.edu.pl; 2 Department of Civil Engineering, NOVA School of Science and Technology, 2829-516 Caparica, Portugal; zdim@fct.unl.pt; 3 IDMEC, Instituto Superior Técnico, Universidade de Lisboa, 1049-003 Lisboa, Portugal

**Keywords:** geosynthetics, geogrid, polyester, recycling of geosynthetics, improved subgrade, recycling of polymers, demolition materials, sustainable development

## Abstract

After years of using geosynthetics in civil engineering and infrastructure construction, it has recently become necessary to consider the possibility of recycling and reusing these materials. This paper presents the results of laboratory tests of the effect of recycled geogrid on the bearing capacity of soils using a CBR test. A polyester geosynthetic was selected for testing due to its high resistance to biodegradation and wide application. In a series of laboratory tests, two types of road and railway subgrade were used, mixed with geosynthetic cuttings in two different weight concentrations. The aim of the research was to demonstrate whether old demolition geosynthetics could be used to strengthen road and rail subgrade as recycled material. The influence of the geosynthetic cutting shape was also considered. The obtained results confirm the possibility of using recycled geogrid to improve the bearing capacity of the pavement subgrade, at least under these laboratory conditions. In the case of sand, the use of 2.0% additive causes that the poorly compacted soil obtains sufficient bearing capacity for the layer of road improved subgrade. As expected, the level of this improvement depends on the type of soil and the shape of geogrid cuttings.

## 1. Introduction

The intensive development of plastics and their utility in everyday life has led to their widespread use around the world. Geosynthetics also belong to the group of materials made of plastics. Currently, they are widely and successfully used in many areas of civil engineering and infrastructure construction [1,2,3,4,5,6,7,8,9,10]. However, after years of using geosynthetics in road and railway applications, it has recently become necessary to consider the possibility of replacing and reusing these materials.

The negative impact of plastics on the environment, due to their resistance to degradation, has already been reported [11]. There are three main methods of handling plastic waste: burying in landfill, incineration and recycling [12,13,14]. Material recycling is currently an important direction for the development of new technologies aimed at protecting the environment and natural resources. Two approaches to polyester recycling are considered profitable: chemical and mechanical processing [15]. It is worth noting that mechanical recycling allows the reutilization of fibers that have already been produced.

According to Monaldo et al. [16], fiber reinforcement is an important process from a structural point of view when developing modern composite materials. In addition, different types of fiber can be used in various civil-engineering applications [16,17,18,19,20,21,22].

During road construction, when dealing with soils with insufficient bearing capacity, it is necessary to prepare a layer of improved subgrade. Such a layer can be prepared by the introduction of a hydraulic binder or by the implementation of a soil/mineral aggregate that is not susceptible to frost heaves [23,24]. The California Bearing Ratio (CBR) of such an unbound soil/aggregate mixture should be at least 20%.

For railway tracks, despite the development of modern technologies, the construction of conventional ballast tracks is still a frequently adopted solution [25]. The use of effective solutions is crucial, considering the increasing rate of climate change. Special attention must be paid to the ballast, track bed and drainage systems in regions where sudden and intense rainfall may occur. Weak soils in the upper part of the track bed demand implementation of a protective layer [26].

According to Gradkowski [17], geofibers obtained from recycled, biologically non-degradable plastics could be considered as good materials for reinforcing the soil layers. Therefore, the use of recycled geofibers in road construction can be treated as one of the pro-ecological technologies aimed at sustainable development. Moreover, soil reinforcement with geofibers can be a cost-effective alternative to soil improvement by hydraulic binder.

**Figure 1:**
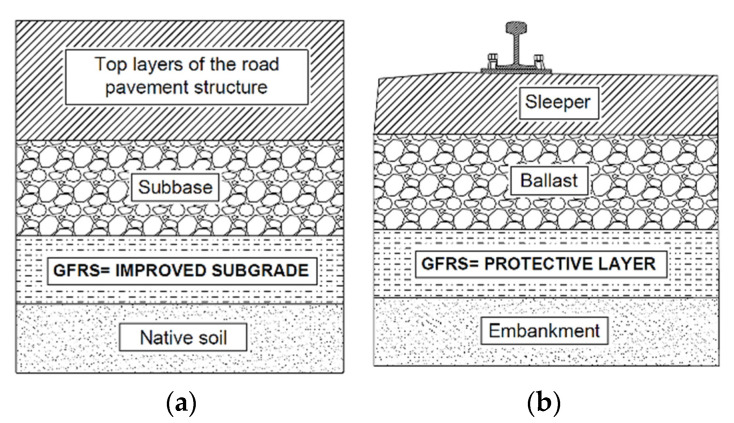
Soil layers where geofibers can be introduced: (**a**) in the layer of improved subgrade under the road pavement structure; (**b**) in the protective layer of railway track.

Examples of locations of the geofiber reinforced soil (GFRS) layer under the road pavement and railway track are shown in Figure 1.

Liu et al. [27] demonstrated the positive impact of fibers on static liquefaction behavior of saturated fiber-reinforced sand in undrained ring-shear tests. Test results indicate that the presence of fibers significantly affects the undrained behavior of medium-dense and dense samples. The results show a continued decrease in soil sample shear resistance after failure, while those treated with fiber show fluctuations even after shear failure. This behavior becomes more evident with increasing fiber content. All the medium-dense and dense reinforced samples maintained structural stability after shearing, while the unreinforced medium-dense sample partly collapsed.

Test results for clay soil presented by Ziegler et al. [28] and Mirzababaei et al. [29] show that fiber inclusion leads to a significant reduction in the number of cracks. Apriyono et al. [30] reported that the application of woven tire waste as soft-clay subgrade reinforcement can significantly improve soil bearing capacity. Similarly, Subash et al. [31] proved that within the scope of their study, the bearing capacity of reinforced ash increases with the increase in geofiber content.

Correia et al. [32] presented slightly different results from the tests of polypropylene fiber effect on the compressive and tensile strength of a soft soil, artificially stabilized with binders. It was stated that a higher quantity of binder improves the mechanical properties of the soil. However, the presence of fibers may reduce this beneficial effect. Moreover, it can be noted that the improvement of the stiffness, compressive and tensile strength is not proportional to the fiber content, but such dependency is non-linear. Nevertheless, Consoli et al. [33] showed that the introduction of fibers increases unconfined compression strength in the cemented soil.

This paper presents the influence of recycled polyester geogrid cuttings on the bearing capacity of soil with the help of the CBR test. Polyester geosynthetic was chosen due to its high resistance to biodegradation and its widespread use. More than 50% of synthetic fibers produced around the world consist of PET. Global consumption of this material has exceeded $ 17 billion per year [11]. Polyester is also used in the production of geosynthetics in road pavements and railway tracks. The geosynthetic market has enormous potential. For example, about 750 km^2^ of geononwovens are produced in the world each year, 60% of which are used in road construction [34]. Thus, the question of geosynthetic recycling is relevant. The aim of the study is to show whether old geosynthetics from demolition can be used to strengthen road and railway subgrade as recycled material.

## 2. Materials and Methods

Laboratory tests were carried out on two types of soils and three fixed levels of soil moisture content. The middle level of moisture content for each soil sample corresponded approximately to the optimum moisture content and the maximum dry density of tested soils. Due to the possibility of a difference between the optimum moisture content of the tested soils and the optimum moisture content of the GFRS, the moisture content of the other samples was deviated by 1.5% above and below this value. Each soil sample was mixed with aged geogrid cuttings to reach 1.0% and 2.0% concentration by weight, resulting in total of 84 samples including the unreinforced ones. The concentration of cuttings was established on the basis of a literature review and the authors’ previous experiences [17,35,36,37]. At such concentrations, an increase in the bearing capacity of the tested soils was observed. GFRS bearing capacity tests were performed on each sample to obtain the CBR.

**Figure 2:**
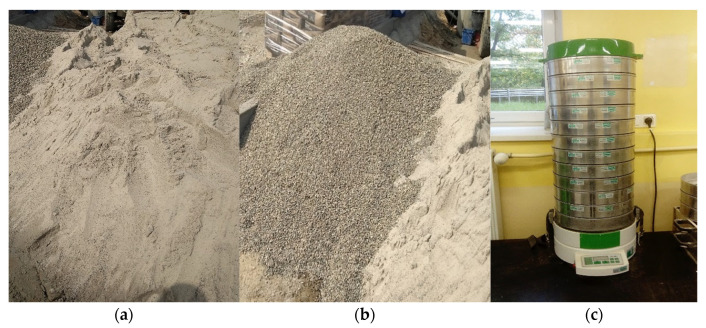
(**a**) Sand used in the laboratory tests; (**b**) gravel and sand used to prepare the sand–gravel mixture; (**c**) set of sieves used to determine the soil gradation curves.

Specifically, two types of natural, non-cohesive soils (sand and sand–gravel mixture) were selected for the laboratory tests. The sand–gravel mixture was made by mixing sand, fine aggregate and gravel to meet the requirements for well-graded material for the road pavement improved subgrade in accordance with Polish guidelines [38]. Soil parameters regarding the grain size were determined using the sieve method according to PN-EN 933-1 [39]. The sand, the sand and gravel used for the mixture and the set of test sieves for the gradation curve determination are shown in Figure 2.

**Figure 3:**
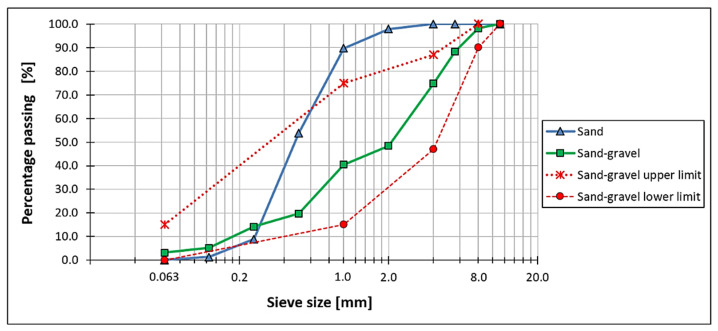
Gradation curve of the tested sand (blue line), gradation curve of the tested sand–gravel mixture (green line), the upper and lower limits for gradation curve of the unbound mineral mixture for the layer of road pavement-improved subgrade (dashed red lines).

The gradation curves of the tested sand and sand–gravel, as well as the boundary curves for well-graded material for road pavement-improved subgrade layer according to the Polish guidelines [38], are shown in Figure 3.

Based on these results, the uniformity coefficient *Cu* and the coefficient of curvature *Cc* were determined according to Equations (1) and (2) [40]:

(1) $$Cu=\frac{D_{60}}{D_{10}}$$

(2) $$Cc=\frac{{\left(D_{30}\right)}^{2}}{D_{10}\cdot D_{60}}$$

where *D*_60_ is the grain diameter (mm) corresponding to 60% passing by weight, *D*_30_ to 30% passing and *D*_10_ to 10% passing.

The following values were obtained: *Cu* = 2.1 and *Cc* = 0.8 for the sand, and *Cu* = 16.3 and *Cc* = 1.1 for the sand–gravel mixture. The results obtained indicate that the sand used is uniformly graded and poorly compacted. On the other hand, the results of the sand–gravel indicate well-graded and well-compacted material [40,41,42].

**Figure 4:**
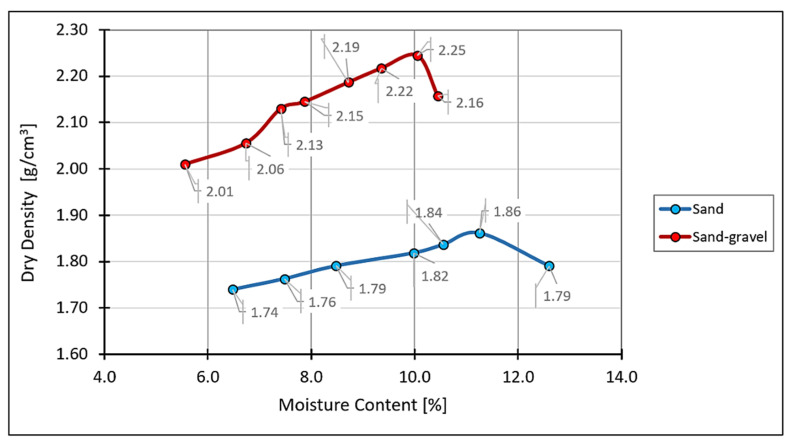
Proctor compaction curve for each soil.

Then, the Proctor method according to PN-EN 13286-2 [43] was used to determine the soil maximum dry density and optimum moisture content. The Proctor compaction test performed on the sand sample showed that the maximum dry density of 1.86 g/cm^3^ was obtained for an optimum moisture content equal to 11.2%. For the sand–gravel, the maximum dry density value of 2.25 g/cm^3^ was obtained for an optimum moisture content equal to 10.0%. The Proctor compaction curves are presented in Figure 4. The selected properties of the tested soils are summarized in Table 1.

According to these results, it was decided to consider for further tests soil moisture of 9.5%, 11.0% and 12.5% for the sand and 8.5%, 10.0% and 11.5% for the sand–gravel. The variants of the tested samples are presented in Table 2 and Table 3.

A biaxial polyester geogrid manufactured from extruded and drawn bars, laid and welded rigidly together [44], was selected as a representative geosynthetic of general use in road and railway engineering. Selected technical data of the geogrid defined in two directions (MD: machine direction, CMD: cross-machine direction) are presented in Table 4 [44].

UV radiation, temperature, chemical and mechanical damage, and treatment are factors significantly affecting the degradation of plastic and geosynthetics [11,45,46,47,48,49,50,51,52,53,54,55,56,57]. At the end of the pavement life, a polyester geosynthetic can be milled using powerful milling machines. During the demolition of old road and railway structures, geosynthetics are mechanically damaged and torn. Solar exposure and cutting were selected to simulate the process of mechanical damage and accelerate aging. The geosynthetic used in laboratory tests was exposed to direct sunlight for a period of 30 days and then subjected to mechanical fragmentation.

**Figure 5:**
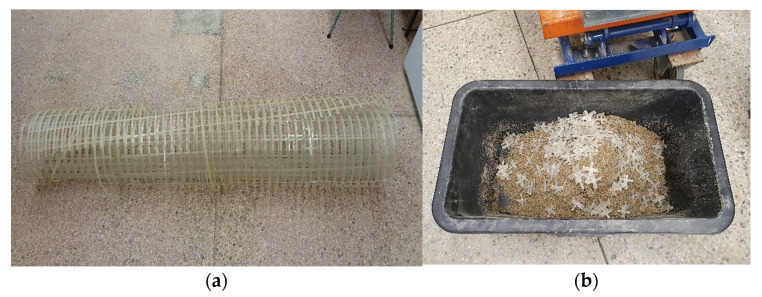
(**a**) Geogrid used in laboratory tests; (**b**) GFRS sample preparation.

The geogrid roll and the GFRS sample preparation are shown in Figure 5.

**Figure 6:**
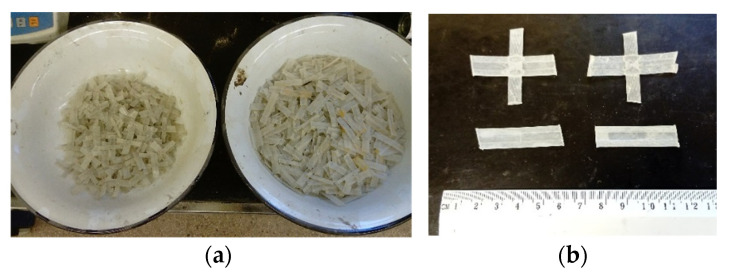
(**a**) Nodes and straight strips prepared to produce a GFRS; (**b**) close-up of the nodes and straight strips.

The geogrid was cut into small pieces. The method of cutting was established so as to obtain straight strips 40 mm long and nodes with orthogonally crossing strips 40 mm long. The length-to-width ratio of the strips was approximately 4. The length of each piece was determined by the aperture size of the geogrid used. Moreover, the length was chosen so that it was the same in the stripes and nodes. Finally, the size of each piece was determined based on the technological ease of mixing the geosynthetic with the soil. The shape of the cut pieces of the geogrid is shown in Figure 6.

**Figure 7:**
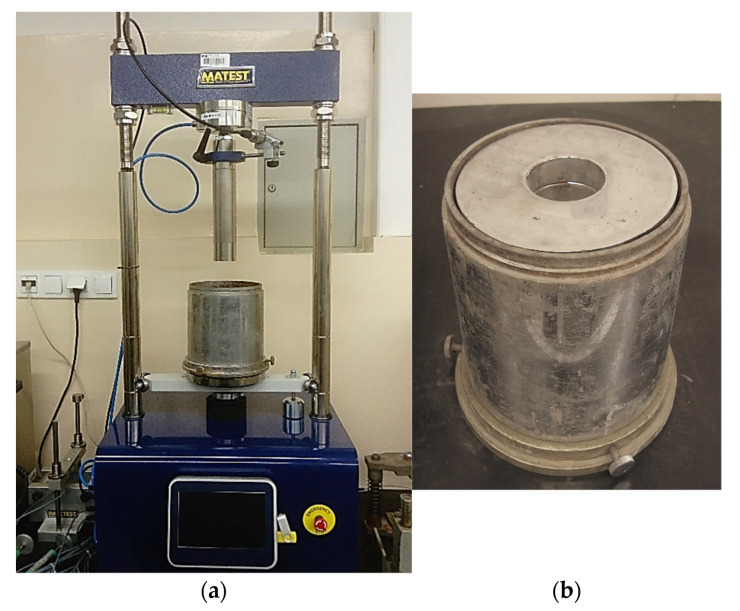
(**a**) Laboratory test stand; (**b**) mold with the sample prepared for testing.

At the last stage, bearing capacity tests (CBR) were carried out according to PN-EN 13286-47 [58] immediately after GFRS compaction. The mold with the sample and the laboratory test stand are shown in Figure 7.

## 3. Results and Discussion

**Figure 8:**
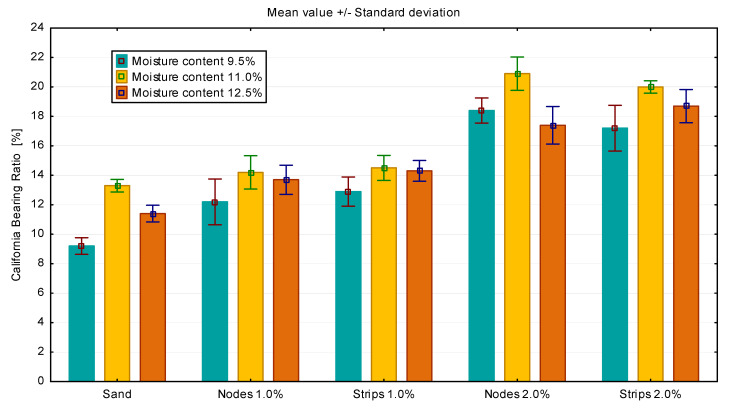
CBR test results for GFRS made of sand.

**Figure 9:**
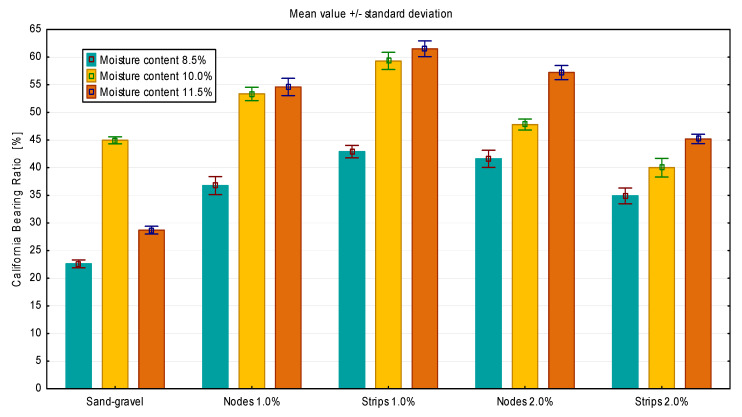
CBR test results for GFRS made of sand–gravel.

The CBR was determined for the prepared GFRS samples. In the case of sand, higher bearing capacity values were obtained for 2.5 mm piston displacement, while in the case of sand–gravel, for 5.0 mm piston displacement. The final results of the bearing capacity tests are shown in Figure 8 and Figure 9.

The results obtained confirmed the positive effect of additives on soil bearing capacity in laboratory tests. For sand with a node content of 2.0%, an increase in soil bearing capacity of about 40% can be observed. The highest load capacities were obtained for humidity close to the optimum soil moisture content. In the case of sand–gravel, the use of 1.0% fibers resulted in the maximum increase in the bearing capacity. For a moisture content of 10.0%, it is about 30%, while for a humidity of 11.5%, more than a double increase in bearing capacity can be observed. However, the use of 2.0% strips caused a decrease in soil bearing capacity at a level of 10% moisture content.

**Figure 10:**
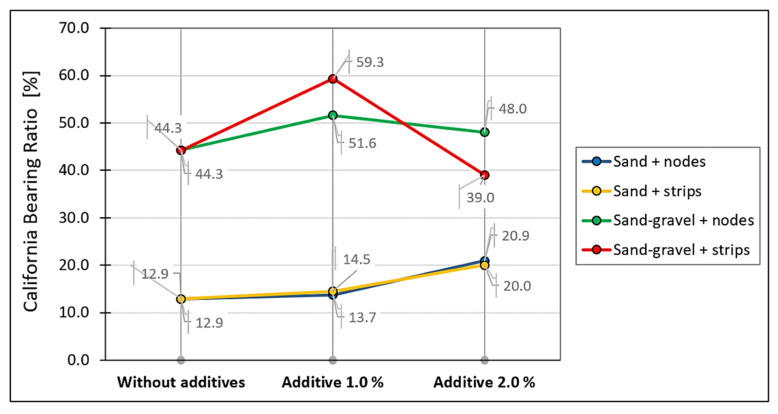
Influence of the additive shape and content on the GFRS bearing capacity at the optimum moisture content of tested soils.

The influence of the additive shape on the bearing capacity of the GFRS at the optimum moisture content of tested soils was also considered. The results are shown in Figure 10.

The influence of the additive shape on the bearing capacity test results of the sand–gravel with strips was noted. The increase in the additive content from 1.0 to 2.0% resulted in a significant decrease in the bearing capacity at the optimum moisture content. In the case of nodes, this decrease was much smaller. In the sand tests, no significant influence of the additive shape on the bearing capacity test results was observed. This difference may be because the sand used is a uniformly graded material. Thus, such results indicate the need for optimization to select the right type and amount of fibers in GFRS layers and the soil gradation curve. The basic mechanism for changing soil properties through the use of recycled geogrid are the additional frictional forces between the soil grains and the geosynthetic pieces. This phenomenon is also described by Gradkowski in [17]. Further research is needed to enable a comprehensive assessment of the performance of mixtures containing recycled geogrid.

## 4. Conclusions

The results of the research allowed us to formulate the following conclusions:

- The use of recycled geosynthetic nodes/strips makes it possible to increase the bearing capacity of soils used in road and railway applications.
- In the case of sand, the use of 2.0% nodes/strips causes the poorly compacted soil with insufficient bearing capacity (CBR < 20%) to obtain sufficient bearing capacity for the layer of road improved subgrade (CBR > 20%).
- In the area covered by tests for sand, a higher content of strips or nodes results in higher bearing capacity. The highest bearing capacity for sand–gravel was obtained in the laboratory tests for 1.0% strip content.
- For the sand–gravel, in the range covered by the study, along with the increase in moisture content, an increase in the bearing capacity of GFRS was observed. The highest bearing capacities were obtained for 11.5% moisture content. This phenomenon was not observed for sand.
- The effect of additive shape was noted in the test of sand–gravel with strips. Increasing the content of the additive to 2.0% resulted in a significant decrease in the bearing capacity. Such an influence of the additive shape on the bearing capacity in the sand tests was not noticed.
- Mechanical treatment can be recognized as an effective method of recycling geosynthetics leading to their reuse. However, the obtained results indicate an obligatory need to optimize the GFRS composition. It is also necessary to check the properties of the GFRS under real field conditions.

## Figures and Tables

**Table 1:** Selected geotechnical properties of the tested soils.

Characteristics	Standard	Sand	Sand–Gravel
Maximum dry density (g/cm^3^)	PN-EN 13286-2	1.86	2.25
Optimum moisture content (%)	PN-EN 13286-2	11.2	10.0
Uniformity coefficient (-)	EN ISO 14688-2	2.1	16.3
Coefficient of curvature (-)	EN ISO 14688-2	0.8	1.1
Sand equivalent (-)	PN-EN 933-8	99	83

**Table 2:** The variants of the tested samples for GFRS made of sand.

Soil + Additive	Additive Weight Concentration (%)	Moisture Content (%)	Number of Samples
sand	0.0	9.5; 11.0; 12.5	6
sand + nodes	1.0	9.5; 11.0; 12.5	9
sand + strips	1.0	9.5; 11.0; 12.5	9
sand + nodes	2.0	9.5; 11.0; 12.5	9
sand + strips	2.0	9.5; 11.0; 12.5	9

**Table 3:** The variants of the tested samples for GFRS made of sand–gravel.

Soil + Additive	Additive Weight Concentration (%)	Moisture Content (%)	Number of Samples
sand–gravel	0.0	8.5; 10.0; 11.5	6
sand–gravel + nodes	1.0	8.5; 10.0; 11.5	9
sand–gravel + strips	1.0	8.5; 10.0; 11.5	9
sand–gravel + nodes	2.0	8.5; 10.0; 11.5	9
sand–gravel + strips	2.0	8.5; 10.0; 11.5	9

**Table 4:** Selected properties of the geogrid defined in two directions.

Characteristics	Standard	MD	CMD
Nominal aperture size (mm)	-	73	30
Nominal tensile strength (kN/m)	EN ISO 10319	80	20
Tensile strength at 2% elongation (kN/m)	EN ISO 10319	28	-
Elongation at nominal tensile strength (%)	EN ISO 10319	≤7	≤7
Construction related strain (%)	-	0	0

## Data Availability

The data presented in this study are available on request from the corresponding author.

## References

[1] Adams M., Ketchart K., Ruckman A., Dimillio A.F., Wu J., Satyanarayana R. (1999). Reinforced Soil for Bridge Support Applications on Low-Volume Roads. Transp. Res. Rec. J. Transp. Res. Board.

[2] Górszczyk J., Grzybowska W. (2011). The use of FEM for thermal analyses of the asphalt pavement. Roads Bridges.

[3] Liu S., Huang H., Qiu T., Kwon J. (2016). Effect of geogrid on railroad ballast particle movement. Transp. Geotech..

[4] Zornberg J.G., Azevedo M., Sikkema M., Odgers B. (2017). Geosynthetics with enhanced lateral drainage capabilities in roadway systems. Transp. Geotech..

[5] Zornberg J.G. (2017). Functions and Applications of Geosynthetics in Roadways. Procedia Eng..

[6] Górszczyk J., Malicki K. (2018). Three-dimensional finite element analysis of geocell-reinforced granular soil. Int. Multidiscip. Sci. GeoConf. SGEM Albena.

[7] Gajewska B., Gajewski M., Kraszewski C. (2018). Analysis of a flexible road pavement on a subgrade with an inclusion in the form of a pipeline running along the road axis. Roads Bridges.

[8] Pires D., Barroso M., Fontul S., Dimitrovová Z. (2018). The study of the interlock of ballast in triaxial geogrids. MATEC Web Conf..

[9] Górszczyk J., Malicki K., Spławińska M. (2018). Structural analysis of soil reinforced by geocell system using analytical-empirical method. Int. Multidiscip. Sci. GeoConf. SGEM.

[10] Górszczyk J., Malicki K. (2019). Digital Image Correlation Method in Monitoring Deformation during Geogrid Testing. Fibres Text. East. Eur..

[11] Webb H.K., Arnott J., Crawford R.J., Ivanova E.P. (2012). Plastic Degradation and Its Environmental Implications with Special Reference to Poly(ethylene terephthalate). Polymers.

[12] Zhang J., Wang X., Gong J., Gu Z. (2004). A study on the biodegradability of polyethylene terephthalate fiber and diethylene glycol terephthalate. J. Appl. Polym. Sci..

[13] Poulikakos L., Papadaskalopoulou C., Hofko B., Gschösser F., Falchetto A.C., Bueno M., Arraigada M., Sousa J., Ruiz R., Petit C. (2017). Harvesting the unexplored potential of European waste materials for road construction. Resour. Conserv. Recycl..

[14] Appiah J.K., Berko-Boateng V.N., Tagbor T.A. (2017). Use of waste plastic materials for road construction in Ghana. Case Stud. Constr. Mater..

[15] Awaja F., Pavel D. (2005). Recycling of PET. Eur. Polym. J..

[16] Monaldo E., Nerilli F., Vairo G. (2019). Basalt-based fiber-reinforced materials and structural applications in civil engineering. Compos. Struct..

[17] Gradkowski K. (2013). Testing ground subgrade reinforced with geosynthetics. Pr. Nauk. Politech. Warsz.-Budownictwo..

[18] Gorino A., Fantilli A.P., Chiaia B. (2017). Optimization of hybrid reinforcement in precast concrete linings using numerical analysis. Roads Bridges-Drog. Mosty.

[19] Radziszewski P., Jackiewicz-Rek W., Sarnowski M., Urbański M. (2018). Fortification of Damaged Asphalt Pavements with Cement Concrete Slabs Reinforced with Next-Gen Bars—Part I: Laboratory Study. Arch. Civ. Eng..

[20] Slebi-Acevedo C.J., Lastra-González P., Pascual-Muñoz P., Castro-Fresno D. (2019). Mechanical performance of fibers in hot mix asphalt: A review. Constr. Build. Mater..

[21] Bompa D., Elghazouli A. (2019). Creep properties of recycled tyre rubber concrete. Constr. Build. Mater..

[22] EsmaeilpourShirvani N., TaghaviGhalesari A., Tabari M.K., Choobbasti A.J. (2019). Improvement of the engineering behavior of sand-clay mixtures using kenaf fiber reinforcement. Transp. Geotech..

[23] Judycki J., Jaskula P., Pszczoła M., Ryś D., Jaczewski M., Alenowicz J., Dołżycki B., Stienss M. (2014). Catalogue of typical flexible and semi-rigid pavements. Gdańsk Univ. Technol. Pol..

[24] Michał Ć., Beata G., Cezary K., Ćwiąkała M., Gajewska B., Kraszewski C., Rafalski L. (2016). Recapitulation of research on frost susceptibility of unbound mixtures for pavement structures. Roads and Bridges Drogi Mosty.

[25] Sañudo R., Miranda M., García C., García-Sanchez D. (2019). Drainage in railways. Constr. Build. Mater..

[26] (2009). Technical Requirements for the Maintenance of the Railway Track-Bed.

[27] Liu J., Wang G., Kamai T., Zhang F., Yang J., Shi B. (2011). Static liquefaction behavior of saturated fiber-reinforced sand in undrained ring-shear tests. Geotext. Geomembr..

[28] Ziegler S., Leshchinsky D., Ling H.I., Perry E.B. (1998). Effect of Short Polymeric Fibers on Crack Development in Clays. Soils Found..

[29] Mirzababaei M., Arulrajah A., Horpibulsuk S., Aldava M. (2017). Shear strength of a fibre-reinforced clay at large shear displacement when subjected to different stress histories. Geotext. Geomembr..

[30] Apriyono A., Sumiyanto, Gusmawan D.D. (2017). Application of Woven Tires Waste as Soft Clay Subgrade Reinforcement for Preventing Highway Structural Failure. AIP Conf. Proc..

[31] Subash S., Mahendran N., Kumar M.M., Nagarajan M. (2017). Performance Analysis of Flexible Pavement with Reinforced Ash. Arch. Civ. Eng..

[32] Correia A.A.S., Oliveira P.J.V., Custódio D.G. (2015). Effect of polypropylene fibres on the compressive and tensile strength of a soft soil, artificially stabilised with binders. Geotext. Geomembr..

[33] Consoli N.C., Bassani M.A.A., Festugato L. (2010). Effect of fiber-reinforcement on the strength of cemented soils. Geotext. Geomembr..

[34] Geomat. https://www.geomatpolska.pl/blog/translate-to-pl-infografika-geotextilie-v-silnicnim-stavitelstvi/.

[35] Stender D. (2020). Laboratory Testing of the Dynamic Modulus of the Geofibre Reinforced Soil. Engineering Thesis.

[36] Fares A. (2019). Preliminary Research on the Effect of Adding Geosynthetic Waste on Selected Strength Parameters of the Sandy Gravel. Master’s Thesis.

[37] Fares R. (2019). Preliminary Research on the Effect of Geosynthetic Waste Addition on Selected Strength Parameters of sand. Master’s Thesis.

[38] Unbound Mixtures for National Roads (2010). Technical Requirements.

[39] PN-EN 933-1 (2012). Tests for Geometrical Properties of Aggregates—Part 1: Determination of Particle Size Distribution-Sieving Method.

[40] Wiłun Z. (2007). Zarys Geotechniki.

[41] PN-EN ISO 14688-2 (2017). Geotechnical Investigation and Testing-Identification and Classification of Soil—Part 2: Principles for a Classification.

[42] PN-EN 933-8 (2012). Tests for Geometrical Properties of Aggregates. Assessment of Fines. Sand Equivalent Test.

[43] PN-EN 13286-2 (2004). Unbound and Hydraulically Bound Mixtures—Part 2: Test Methods for the Determination of Laboratory Reference Density and Water Content—Proctor Compaction.

[44] NAUE Geosynthetics. http://www.naue.us/products/secugrid/.

[45] Rowe R., Rimal S., Sangam H. (2008). Ageing of HDPE geomembrane exposed to air, water and leachate at different temperatures☆. Geotext. Geomembr..

[46] Kiersnowska A. (2016). Accelerated aging tests in assessment of the durability geogrid polymer used to reinforce the slope on landfill. Acta Sci. Pol. Arch..

[47] Sołkowski J., Górszczyk J., Malicki K., Kudła D. (2021). The Effect of Fatigue Test on the Mechanical Properties of the Cellular Polyurethane Mats Used in Tram and Railway Tracks. Materials.

[48] Zhao Y., Ling X., Gong W., Li P., Li G., Wang L. (2020). Mechanical Properties of Fiber-Reinforced Soil under Triaxial Compression and Parameter Determination Based on the Duncan-Chang Model. Appl. Sci..

[49] Hufenus R., Rüegger R., Flum D., Sterba I.J. (2005). Strength reduction factors due to installation damage of reinforcing geosynthetics. Geotext. Geomembr..

[50] Vieira C.S., Pereira P.M. (2021). Short-term tensile behaviour of three geosynthetics after exposure to Recycled Construction and Demolition materials. Constr. Build. Mater..

[51] Vieira C.S., Lopes M.D.L. (2016). Damage Induced by Recycled Aggregates on the Short-Term Tensile Behaviour of a High-Strength Geotextile. Procedia Eng..

[52] Valipour M., Shourijeh P.T., Mohammadinia A. (2021). Application of recycled tire polymer fibers and glass fibers for clay reinforcement. Transp. Geotech..

[53] Rahman T., Mohajerani A., Giustozzi F. (2020). Recycling of Waste Materials for Asphalt Concrete and Bitumen: A Review. Materials.

[54] Xiao Y., Erkens S., Li M., Ma T., Liu X. (2020). Sustainable Designed Pavement Materials. Materials.

[55] Elsing A. (2005). Experience from more than 30 years of asphalt reinforcement with polyester grids. Proceedings of the Seventh International Conference on the Bearing Capacity of Roads, Railways and Airfields.

[56] Górszczyk J., Malicki K. (2020). Experimental investigation of loading frequency influence on a strength of asphalt interlayer bonding. Arch. Civ. Eng..

[57] Górszczyk J., Malicki K. (2017). Study of the mechanical properties of a hexagonal geogrid using the digital image correlation method. Int. Multidiscip. Sci. GeoConf. SGEM.

[58] PN-EN 13286-47 (2012). Unbound and Hydraulically Bound Mixtures—Part 2: Test Methods for the Determination of California Bearing Ratio, Immediate Bearing Index and Linear Swelling.

